# Extensive Von Meyenburg Complexes Mimicking Diffuse Hepatic Metastases: Diagnostic Role of MRI and Magnetic Resonance Cholangiopancreatography

**DOI:** 10.7759/cureus.110832

**Published:** 2026-06-14

**Authors:** Momin Masued, Neeraj Vinayakumar, Aamna Ahmed

**Affiliations:** 1 Internal Medicine, The Princess Alexandra Hospital NHS Trust, Harlow, GBR; 2 Gastroenterology, The Princess Alexandra Hospital NHS Trust, Harlow, GBR; 3 Pharmacy, The Princess Alexandra Hospital NHS Trust, Harlow, GBR

**Keywords:** biliary hamartoma, hepatic metastases mimic, liver imaging, liver lesions, mrcp, von meyenburg complexes

## Abstract

Von Meyenburg complexes (VMCs), also known as biliary hamartomas, are benign ductal plate malformations that are frequently identified incidentally on imaging. When numerous and diffusely distributed throughout the liver, they may mimic metastatic disease and create significant diagnostic uncertainty.

We report the case of a woman in her fifties who presented with abdominal pain and underwent contrast-enhanced computed tomography (CT), which demonstrated multiple low-attenuation lesions throughout both hepatic lobes. This distribution pattern raised concern for diffuse hepatic metastatic disease despite the absence of a known primary malignancy. Subsequent magnetic resonance cholangiopancreatography (MRCP) demonstrated innumerable subcentimetre T2-hyperintense lesions scattered throughout both hepatic lobes, without communication with the biliary tree, a dominant hepatic mass, or suspicious solid components. These appearances were characteristic of von Meyenburg complexes and inconsistent with diffuse metastatic disease. The case was reviewed at an Upper Gastrointestinal Multidisciplinary Team (UGI MDT) meeting, where the lesions were considered benign and no further invasive investigation was required.

This case highlights the importance of recognising the characteristic MRI features of VMCs. Accurate radiological diagnosis can facilitate confident non-invasive diagnosis, avoid unnecessary invasive procedures and inappropriate oncological referral, and reduce anxiety associated with a suspected diagnosis of metastatic malignancy.

## Introduction

Von Meyenburg complexes (VMCs), also known as biliary hamartomas, are benign congenital malformations of the intrahepatic bile ducts resulting from incomplete involution of embryological ductal plate structures [1]. They belong to the spectrum of ductal plate malformations and are generally identified incidentally during imaging, surgery, or autopsy. Although usually clinically insignificant, extensive disease may present a substantial diagnostic challenge because the radiological appearances can closely mimic diffuse hepatic metastases.

The prevalence of VMCs has been estimated at approximately 0.6-5.6% in autopsy studies [2,3]. Histologically, they consist of irregularly dilated bile duct remnants lined by cuboidal epithelium and embedded within fibrous stroma [2]. The fluid content of these lesions accounts for their characteristic MRI appearance, including high signal intensity on T2-weighted imaging.

Most lesions are small, measuring less than 15 mm, and are distributed throughout the hepatic parenchyma [2]. While patients are typically asymptomatic, increasing utilisation of cross-sectional imaging has led to more frequent recognition of these lesions [3,4]. Their identification is clinically important because misinterpretation may prompt unnecessary investigations, including tumour marker testing, repeated imaging, biopsy, or referral to oncological services [2].

Computed tomography (CT) frequently demonstrates multiple small hypoattenuating lesions, although appearances may be non-specific. Magnetic resonance imaging (MRI), particularly magnetic resonance cholangiopancreatography (MRCP) sequences, is often diagnostic because lesions characteristically demonstrate T2 hyperintensity and lack communication with the biliary tree. Recognition of these features is essential when differentiating VMCs from metastatic disease, simple hepatic cysts, Caroli disease, polycystic liver disease, biliary cystic neoplasms, microabscesses, and other cystic hepatic lesions [3-5].

We present a case of extensive VMCs initially suspected to represent diffuse hepatic metastatic disease on CT, highlighting the pivotal role of MRI and MRCP in establishing a confident non-invasive diagnosis and avoiding unnecessary oncological investigation.

## Case presentation

A woman in her fifties presented with a two-week history of intermittent sharp abdominal pain associated with nausea and a single episode of vomiting. She denied weight loss, jaundice, anorexia, fevers, night sweats, or other constitutional symptoms. Her medical history included hypothyroidism and depression. She was a non-smoker and reported minimal alcohol consumption.

Clinical examination demonstrated mild epigastric tenderness without signs of peritonism or chronic liver disease. Initial laboratory investigations, including liver function tests and inflammatory markers, were within normal limits.

Contrast-enhanced CT of the abdomen and pelvis demonstrated multiple well-defined low-attenuation lesions distributed throughout both hepatic lobes (Figure 1).

**Figure 1:**
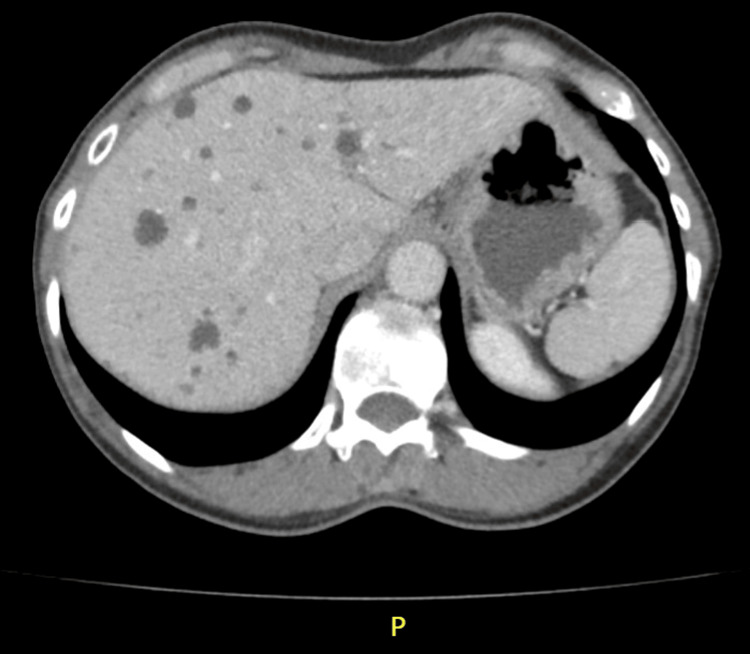
Contrast-enhanced CT of the liver Axial contrast-enhanced CT image demonstrating multiple well-defined hypoattenuating lesions distributed throughout both hepatic lobes. Their multiplicity and diffuse distribution prompted further evaluation to exclude metastatic disease. No overt aggressive imaging features are identified.

The multiplicity and diffuse bilateral distribution of the lesions prompted further evaluation to exclude metastatic disease, although multiple biliary hamartomas and hepatic cysts were also considered in the differential diagnosis. No primary malignancy was identified on CT. The lesions were well circumscribed and showed no associated suspicious enhancing soft tissue components or other overt aggressive imaging features. Mild pancreatic duct dilatation was noted without evidence of a pancreatic mass or biliary obstruction.

Further evaluation was performed using magnetic resonance cholangiopancreatography (MRCP). This demonstrated innumerable small T2-hyperintense lesions scattered throughout the liver parenchyma without communication with the biliary tree (Figures 2, 3).

**Figure 2:**
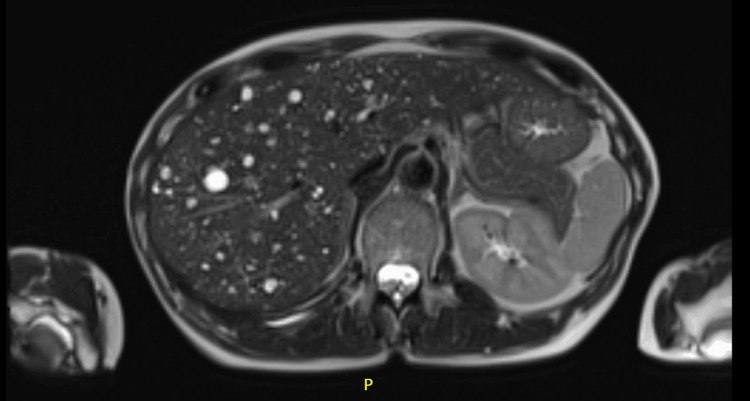
Axial T2-weighted MRI of the liver Axial T2-weighted image demonstrating numerous small hyperintense lesions throughout the hepatic parenchyma, producing the characteristic “starry-sky” appearance associated with von Meyenburg complexes.

**Figure 3:**
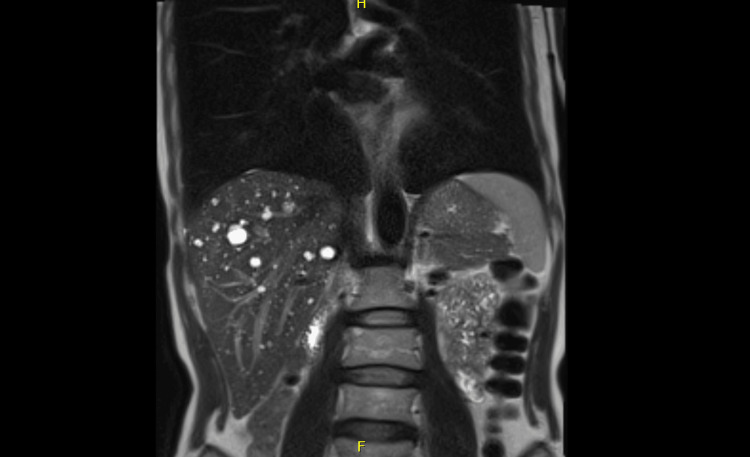
Coronal T2-weighted MRI of the liver Coronal T2-weighted image demonstrating diffuse distribution of multiple T2-hyperintense lesions throughout both hepatic lobes, consistent with biliary hamartomas.

Corresponding lesions demonstrated low signal intensity on T1-weighted imaging (Figure 4).

**Figure 4:**
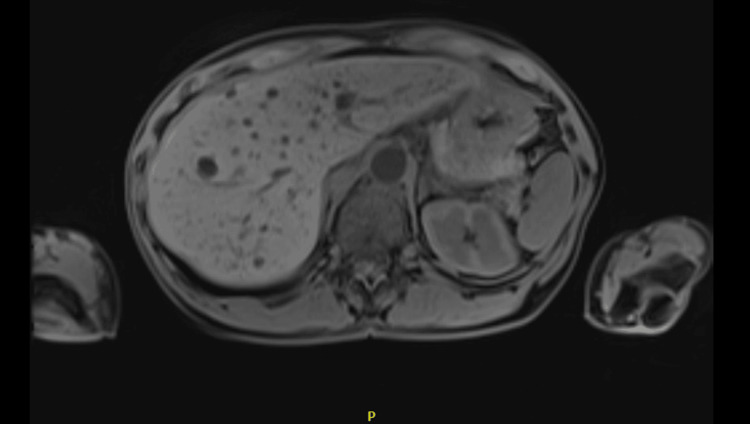
Axial T1-weighted MRI of the liver Axial T1-weighted image demonstrating corresponding low-signal hepatic lesions, supporting the diagnosis of von Meyenburg complexes.

The intrahepatic and extrahepatic bile ducts were otherwise normal in calibre and appearance. No dominant hepatic mass, suspicious solid component, biliary obstruction, or other features suggestive of malignancy were identified. These findings were considered characteristic of extensive VMCs.

The case was subsequently discussed at an Upper Gastrointestinal Multidisciplinary Team (UGI MDT) meeting. Following multidisciplinary review of the CT and MRI findings, the lesions were considered characteristic of extensive VMCs. No evidence of upper gastrointestinal malignancy was identified and no biopsy or further invasive investigation was recommended. The patient was subsequently discharged from the upper gastrointestinal cancer pathway.

## Discussion

VMCs, also known as biliary hamartomas, are benign ductal plate malformations resulting from incomplete embryological remodelling of the intrahepatic biliary system [1]. Although typically identified incidentally and of little clinical significance, extensive disease may represent a considerable diagnostic challenge because the imaging appearances can closely mimic diffuse hepatic metastatic disease [2,3].

On CT, VMCs usually appear as multiple small hypoattenuating lesions distributed throughout the liver parenchyma [4]. However, CT findings are often non-specific and may be insufficient for definitive characterisation [6]. In the absence of prior imaging or histological confirmation, extensive disease may generate understandable concern for disseminated malignancy, particularly when lesions are numerous and involve both hepatic lobes [6,7]. This can result in additional investigations, referral to specialist cancer pathways, and significant patient anxiety [6,7].

MRI provides the most valuable non-invasive modality for lesion characterisation [4,7]. Classically, VMCs demonstrate low signal intensity on T1-weighted imaging and marked hyperintensity on T2-weighted imaging owing to their fluid content [8]. Lesions typically do not communicate with the biliary tree, an important distinguishing feature from Caroli disease [9]. The characteristic “starry-sky” appearance observed in this case has been repeatedly described in the radiological literature and is highly suggestive of VMCs when identified in the appropriate clinical context [4,5].

The differential diagnosis includes metastatic disease, hepatic microabscesses, Caroli disease, simple hepatic cysts, polycystic liver disease, biliary cystic neoplasms, and other diffuse cystic liver disorders [5,6-9]. Hepatic metastases may demonstrate variable enhancement patterns, interval growth, diffusion restriction, or association with a known primary malignancy [5]. Hepatic microabscesses are usually accompanied by systemic symptoms and elevated inflammatory markers. Caroli disease is characterised by segmental dilatation of intrahepatic bile ducts with demonstrable communication with the biliary tree [9]. Polycystic liver disease typically produces larger cystic lesions and is often associated with renal cystic disease. Biliary cystic neoplasms are generally larger, more complex lesions that may demonstrate septations, mural nodularity, or enhancement [5]. 

Several imaging features favour a diagnosis of VMCs, including small lesion size (typically less than 15 mm), diffuse distribution throughout the hepatic parenchyma, uniform T2 hyperintensity, corresponding T1 hypointensity, absence of significant mass effect, lack of suspicious enhancing soft tissue components, and absence of communication with the biliary tree [4,5,8]. Recognition of these imaging characteristics can help radiologists distinguish VMCs from more sinister hepatic pathology and avoid unnecessary invasive investigations.

The distinction is particularly important in patients with a known primary malignancy, where extensive VMCs may be misinterpreted as diffuse hepatic metastatic disease [2]. Such misclassification has the potential to alter tumour staging, prognosis, treatment options, and eligibility for curative intervention. Familiarity with the characteristic MRI appearances is therefore essential for radiologists and MDTs involved in the assessment of indeterminate hepatic lesions.

Although VMCs are overwhelmingly benign lesions, rare cases of malignant transformation to cholangiocarcinoma have been reported in the literature [2,10]. Biopsy may therefore be considered when imaging appearances are atypical, lesions demonstrate interval growth, suspicious enhancement characteristics are present, or clinical findings are discordant with a benign diagnosis [2,5]. In patients with characteristic MRI findings and multidisciplinary consensus, however, histopathological confirmation is often unnecessary.

Histopathological confirmation was not obtained in this case because the imaging findings were considered characteristic of VMCs following multidisciplinary review. Although diffusion-weighted imaging and longitudinal imaging follow-up were unavailable, the combination of characteristic MRCP appearances, absence of biliary communication, and multidisciplinary consensus provided sufficient diagnostic confidence to establish a non-invasive diagnosis [4].

In the present case, MRI and MRCP provided sufficient diagnostic confidence to avoid biopsy, exclude upper gastrointestinal malignancy at multidisciplinary review, and permit discharge from the cancer pathway without further invasive investigation. This case highlights the pivotal role of MRI and MRCP in characterising indeterminate hepatic lesions and demonstrates how recognition of benign radiological mimics can prevent inappropriate oncological referral and unnecessary patient distress [7].

## Conclusions

VMCs should be considered in the differential diagnosis of multiple hepatic lesions, particularly when MRI and MRCP demonstrate numerous small T2-hyperintense lesions without communication with the biliary tree. Recognition of these characteristic imaging features can facilitate confident non-invasive diagnosis and help distinguish benign biliary hamartomas from diffuse hepatic metastatic disease. MRI and MRCP play pivotal roles in lesion characterisation when CT findings are indeterminate. Accurate radiological interpretation may prevent unnecessary invasive investigations, inappropriate oncological referral, and associated patient anxiety. Awareness of this uncommon entity is therefore important for radiologists and MDTs involved in the assessment of diffuse hepatic lesions.
